# Screen time and cardiometabolic function in Dutch 5–6 year olds: cross-sectional analysis of the ABCD-study

**DOI:** 10.1186/1471-2458-14-933

**Published:** 2014-09-08

**Authors:** Mai JM Chinapaw, Teatske M Altenburg, Manon van Eijsden, Reinoud JBJ Gemke, Tanja GM Vrijkotte

**Affiliations:** Department of Public and Occupational Health and the EMGO nstitute for Health and Care Research, VU University Medical Center, Van der Boechorststraat 7, Amsterdam, 1081 BT The Netherlands; Department of Epidemiology Documentation and Health Promotion, Public Health Service, Amsterdam, The Netherlands; Department of Paediatrics, the EMGO Institute for Health and Care Research, VU University Medical Center, Amsterdam, The Netherlands; Department of Public Health, Academic Medical Centre, University of Amsterdam, Amsterdam, The Netherlands; Department of Health Sciences, VU University, Amsterdam, The Netherlands

**Keywords:** Children, Sitting, Weight status, Metabolic syndrome

## Abstract

**Background:**

Evidence on the association between different screen behaviours and cardiometabolic biomarkers in children is limited. We examined the independent relationship of TV time and PC time with cardiometabolic biomarkers in Dutch 5–6 year old children.

**Methods:**

Cross-sectional analyses were conducted December 2012-March 2013 using data from a multi-ethnic cohort (the ABCD study, n = 1,961). TV and PC time and physical activity were assessed by parent-report. Body weight, height, waist circumference and blood pressure were measured using a standard protocol. Fasting capillary blood samples were collected. A cardiometabolic function score was computed as the mean of the inverted standardised values of waist circumference, mean of systolic and diastolic blood pressure, glucose, HDLC (not inverted), and triglycerides.

**Results:**

Mean TV time was 1.2 (±0.8) hr/day and mean PC time was 0.2 (±0.4) hr/day. After adjustment for birth weight, height, maternal education, PC time, and physical activity, excessive TV time (>2 hrs/day) was adversely associated with waist circumference (b = 0.39, 95% CI: 0.004;0.78) while PC time was beneficially associated with HDLC levels (b = 0.04, 95% CI: 0.001;0.08). We found no additional significant associations of TV time, or PC time with any of the cardiometabolic biomarkers.

**Conclusions:**

We found no convincing evidence for an association between TV or PC time and cardiometabolic function in apparently healthy 5–6 yr olds.

## Background

Children spend a substantial amount of their time with electronic media – TV, videogames and Internet [1]. Highly prevalent sedentary behaviours include TV viewing and PC use. A recent European study showed that European 10–12 yr olds spent around 2 to 2.5 hours per day on screen-viewing activities (i.e., time sitting in front of a TV or computer screen or playing video games) [2]. In the Longitudinal Study of Australian Children, 90% of 4–5 year olds spend more than two hours per day on screen-viewing activities [3].

In adults, there is a growing body of evidence that sedentary behaviour may be a distinct risk factor for multiple adverse health outcomes, including cardiometabolic biomarkers, type 2 diabetes and premature mortality [4, 5]. Importantly, these detrimental associations remain even after accounting for time spent in leisure time physical activity [6]. However, since most studies adjusted only for self-reported physical activity, there is scope for residual confounding. The American Academy of Pediatrics [7] and the Australian department of Health and Aging [8] recommend that children younger than 2 years of age avoid all types of TV viewing and that children 2 years of age or older should not be exposed to more than 1–2 hours per day of screen time. However, up to now, there is insufficient evidence for a prospective relationship between screen time and health indicators, except for moderate evidence for an association with aerobic fitness [9, 10]. Moreover, few studies examined the association between screen time and various cardiometabolic health indicators especially in children [9, 11–13]. Evidence on such an association in children is important as cardiometabolic risk factors track from childhood to adulthood [14] in addition, these risk factors reinforce each other resulting in the development of type 2 diabetes and cardiovascular disease [15].

Therefore, we aimed to examine the cross-sectional association between parent reported TV time and PC time, independently from physical activity, and cardiometabolic function in a sample of 5–6 year-old Dutch children.

## Methods

The present study is part of the Amsterdam Born Children and their Development (ABCD) study (http://www.abcd-study.nl), a population-based prospective cohort study that examines the relationship of maternal lifestyle and psychosocial determinants during pregnancy with multiple aspects of child development and health [16]. Approval of the study was obtained from the Academic Medical Center Medical Ethical Committee, the VU University Medical Center Medical Ethical Committee and the Registration Committee of Amsterdam. All participating mothers gave written informed consent for themselves and their children.

### Participants

Between January 2003 and March 2004, pregnant women in Amsterdam were asked to participate in the ABCD study during their first prenatal visit to an obstetric care provider. In total 12,373 women were approached; by estimate 99% of the target population [16]. A questionnaire covering socio-demographic characteristics, obstetric history, lifestyle, and psychosocial conditions was sent to the women’s home addresses and the women were requested to return it to the Public Health Service by prepaid mail. Of these 12,373 approached women, 8,266 women filled in the pregnancy questionnaire (response rate 67%). A total of 6,735 (81%) women gave permission for follow-up. In the following years, growth data of the children were collected from Child Health Care centres. In 2008, when the children turned five, the addresses of 6,161 mothers were retrieved from the Child Health Care registry; attrition in this follow-up number was largely due to untraceable changes in address or migration. The mothers received a questionnaire, including an informed consent sheet for a health check of their children at age 5. Children were invited for the health check at school for children living in Amsterdam, at a central location in the city for participants living outside Amsterdam until December 2010. The health check included (amongst others) a capillary blood sample (n = 2,108 correct samples) and anthropometrics (n = 3,321), for which separate consents were signed. The questionnaire (n = 4,488) encompassed items about the child’s demography, health status (consultations and medication), nutrition (qualitative and quantitative aspects), physical activity, screen time and familiar risk factors for cardiovascular diseases. Trained personnel from the Public Health Service Amsterdam performed all measurements. Only children with complete data on cardiometabolic biomarkers and screen time were included in the present analyses (n = 1,961) conducted in December 2012-March 2013. Participants with measured fasting blood showed a higher proportion of Dutch ethnicity, lower systolic BP and diastolic BP but no difference in mean age, gender, BMI and WC than participants without fasting blood measurement [17].

### Measures at age 5

#### Demographics

Information on child’s sex, date of birth, and birth weight was gathered from the Child Health Care registry. Maternal educational level (highest level of education) and ethnicity (based on country of birth of the mother) was gathered by questionnaire.

### Cardiometabolic biomarkers

Trained research assistants measured weight, height, waist circumference (WC), and systolic and diastolic blood pressure using standard protocols. Height was measured to the nearest millimetre using a Leicester portable height measure (Seca, Hamburg, Germany) and weight to the nearest 100 gram using a Marsden MS-4102 weighing scale (Oxfordshire, United Kingdom). Height and weight were measured in order to calculate Body Mass Index (BMI) (kg/m^2^). Children were classified as normal weight, overweight or obese according to age and gender-specific cut-off points [18]. WC was measured to the nearest millimetre midway between the costal border and iliac crest, using a Seca measuring tape. For the blood pressure measurements the child first lied down in a supine position. During the first minute, one test blood pressure measurement was performed. Then the child lied in rest for four minutes. Next the child was seated at a table and acclimatized for one minute, followed by four minutes of sitting in rest. Finally, systolic and diastolic blood pressure was measured twice in sitting position [19]. Systolic and diastolic blood pressure (mmHg) were calculated by taking the mean value of the two measures. Using a finger puncture, overnight-fasting capillary blood samples (0.5 ml) were collected to determine glucose, total cholesterol, low density lipoprotein cholesterol (LDLC), high density lipoprotein cholesterol (HDLC), and triglycerides [Demecal, Lab Anywhere, The Netherlands] [20]. The parents were asked to have their child fast from the evening before the morning of blood sampling. The fasting samples were taken between 8:00 and 8:30 A.M. and the children were offered breakfast afterwards.

### Screen time

Mothers were asked to record the time that their child spent watching TV or DVD at home or at friends’ homes on a 7-point scale ranging from almost never to 5 hours/day or more for weekdays and weekend days separately (1 = (almost) never, 2 = <1 hour/day, 3 = 1 hour/day, 4 = 2 hours/day, 5 = 3 hours/day, 6 = 4 hours/day, 7 = 5 hours/day). Responses to each were recorded and summed to compute average hours/day of TV time as follows: (Average TV time on a weekday*5 + Average TV time on a weekend day*2) / 7, possible range 0–5. Additionally, TV time was dichotomised according to screen time recommendations for children i.e. <2 hours/day. The time that their child spent using the PC or game console at home or at friends’ homes was asked and recorded on the same 7-point scale for weekdays and weekend days separately. Responses to each were averaged into hours/day of PC time (possible range 0–5). Since few children used the PC for more than 2 hours/day this variable was not dichotomized.

### Physical activity (PA)

PA questions included duration of playing outside in summer and winter for weekdays and weekend days separately (0–5 hours/day), sports participation (0–4 hours/week), and frequency of walking and biking to and from school (0–5 times/week). Responses to playing outside in summer and winter were averaged to compute hours/day playing outside. Mean duration (hours/day) playing outside (0–5), mean duration (hours/week) sports participation (0–4) and frequency (times/week) of walking and biking to school (0–5) were averaged into a PA score with higher scores indicating higher levels of PA.

### Data analysis

A relative cardiometabolic function score was computed as the mean of the standardised scores from the following five variables: WC, mean of systolic and diastolic blood pressure, glucose, HDLC, and triglycerides [11, 13]. Each of these variables was first standardised (individual value – group mean)/standard deviations (SD), stratified by gender. All standardised values, except for HDLC, were multiplied by −1 to confer enhanced function with increasing values for the purpose of calculating the metabolic function score. Using a continuously distributed sum score makes more sense in a sample of young and healthy children, since prevalence of deviating values will be low. Moreover, using a continuously distributed sum score maximizes statistical power. Descriptive results are presented as mean (±standard deviation) for continuous variables and percentages for categorical variables. Gender differences in cardiometabolic biomarkers, screen time, and PA were assessed by independent t-tests.

The relationship between screen time and the cardiometabolic biomarkers/function score was examined using linear regression analysis adjusting for gender (except when the gender-specific function score was the outcome), birth weight [21], and maternal education (model 1). To examine the independent association of screen time and cardiometabolic biomarkers in a subsequent model, adjustments for TV time (except when TV time was the exposure), PC time (except when PC time was the exposure), PA, and WC (except when the cardiometabolic function score or WC was the outcome) were made (model 2). In both models the analyses with WC, blood pressure and metabolic function score as outcome, were additionally adjusted for height since at this age height is related to WC and blood pressure. Additionally, the independent association of excessive TV time (defined as 2 hours or more per day) and cardiometabolic biomarkers was examined, adjusted for PC time, PA, and WC (except when the cardiometabolic function score or WC was the outcome). Significance levels were set at p ≤ .05.

## Results

Table 1 presents the characteristics of the participants. 91.5% of the children were categorised as normal weight, 7% were overweight, and 1.5% were obese. Table 2 presents time spent on TV viewing and PC use, PA and the cardiometabolic biomarkers/function score. The mean time spent TV viewing was 1.2 hour per day and mean PC use was 0.2 hours per day. Boys had significantly higher screen times than girls. Girls had significantly higher diastolic blood pressure, LDLC and triglyceride levels than boys, while their WC and glucose levels were significantly lower.

**Table 1:** **Characteristics (means ± standard deviation) of 1,961 Dutch 5-6 yr old children**

	Boys N = 1,000	Girls N = 961
Age (yr)	5.6 (±0.4)	5.6 (±0.4)
Height (m)	116.5 (±5.5)	115.8 (±5.5)
Weight (kg)	21.1 (±2.9)	20.8 (±3.3)
BMI (kg/m^2^)	15.5 (±1.3)	15.5 (±1.5)
% overweight	6	8
% obese	1	2
Ethnicity (% non-Western)	16%	15%
Birth weight	3575 (±556)	3431 (±524)
Maternal education (% higher education)	70%	69%

BMI = Body Mass Index.

**Table 2:** **Screen time, physical activity, cardiometabolic biomarkers (means ± SD) of 1,961 Dutch 5-6 yr old children**

	Boys N = 1,000	Girls N = 961
Playing outside (0–5 hrs/day)	1.9 (±0.8)	1.9 (±0.7)
Walking to school (%)		
• Never	43%	41%
• 1-4×/wk	34%	36%
• 5×/wk	23%	23%
Biking to school (%)		
• Never	65%	67%
• 1-4×/wk	30%	29%
• 5×/wk	5%	4%
Sports (hrs/wk, possible range: 0–4)	0.6 (±0.8)	0.7 (±0.8)*
PA score (possible range: 0–4)	1.3 (±0.6)	1.3 (±0.6)
TV/DVD/video time (hrs/day, possible range: 0–5)	1.2 (±0.8)	1.1 (±0.8)*
Computer and electronic games (hrs/day possible range: 0–5)	0.3 (±0.5)	0.2 (±0.4)*
WC (cm)	52.8 (±3.4)	51.9 (±3.7)*
Systolic blood pressure (mm Hg)	98.6 (±8.5)	97.8 (±8.4)
Diastolic blood pressure (mm Hg)	58.3 (±8.4)	59.7 (±8.1)*
Glucose (mmol/l)	4.6 (±0.5)	4.5 (±0.4)*
LDLC (mmol/l)	2.2 (±0.6)	2.4 (±0.7)*
HDLC (mmol/l)	1.3 (±0.3)	1.3 (±0.3)
Tiglycerides (mmol/l)	0.6 (±0.3)	0.7 (±0.3)*
Cardiometabolic function score (z-score)^a^	0.0 (±0.5)	0.0 (±0.5)

*p < 0.05.

LDLC = low-density lipoprotein cholesterol, HDLC = high-density lipoprotein cholesterol, WC = waist circumference.

^a^Higher values indicating better cardiometabolic function.

Table 3 shows the separate associations between TV time and PC time with the cardiometabolic biomarkers/function score. In the fully adjusted model we found no significant associations between TV time (hr/day) and cardiometabolic biomarkers, but excessive TV time (>2 hrs/day) was significantly associated with waist circumference (b = 0.39, 95% CI: 0.004;0.78). In the fully adjusted model PC time was significantly positively associated with HDLC levels (b = 0.04, 95% CI: 0.001;0.08). This means that with each hour PC viewing HDLC levels increased by 0.04 mmol/l.

**Table 3:** **Associations of TV time and PC time with cardiometabolic biomarkers in Dutch 5**-**6 yr old children (N = 1,961)**

	TV time(b-coefficient [95%CI])		PC time(b-coefficient [95%CI])	
	Model 1	Model 2	Model 1	Model 2
WC (cm)	0.18 (−0.03;0.40)	0.09 (−0.13;0.30)	0.03 (−0.34;0.41)	−0.05 (−0.41;0.32)
Mean blood pressure (mm Hg)	0.43 (−0.04;0.91)	0.34 (−0.16;0.83)	0.50 (−0.32;1.31)	0.32 (−0.53;1.18)
Glucose (mmol/l)	0.04 (0.004;0.07)*	0.02 (−0.01;0.06)	0.07 (0.01;0.12)*	0.05 (−0.01;0.11)
LDLC (mmol/l)	0.03 (−0.02;0.07)	0.02 (−0.02;0.07)	0.04 (−0.04;0.11)	0.02 (−0.06;0.10)
HDLC (mmol/l)	0.01 (−0.01;0.03)	0.001 (−0.02;0.02)	0.04 (0.004;0.08)*	0.04 (0.001;0.08)*
Triglycerides (mmol/l)	0.002 (−0.02;0.02)	0.002 (−0.02;0.02)	−0.003 (−0.04;0.03)	−0.004 (−0.04;0.03)
Cardiometabolic function score^a^	0.02 (−0.01;0.05)	0.02 (−0.01;0.05)	−0.001 (−0.05;0.05)	−0.01 (−0.07;0.04)

*p < 0.05.

^a^Higher values indicating better cardiometabolic function, *p < 0.05.

LDLC = low-density lipoprotein cholesterol, HDLC = high-density lipoprotein cholesterol, WC = waist circumference.

Data are unstandardised regression coefficients (95% CI) and outcomes are expressed as standardised z scores.

Model 1: adjusted for gender (except when the gender-specific function score was the outcome), birth weight, and maternal education. Model 2: Model 1 + additionally adjusted for TV time (except when TV time as exposure), PC time (except when PC time as exposure), PA, and WC (except when function score and WC as outcome). In both models the analyses for WC, blood pressure and function score were also adjusted for child height.

## Discussion

In this population-based sample of Dutch 5–6 yr olds, TV time and PC time were not significantly associated with the cardiometabolic function score, independent of PA and other potential confounding variables. The lack of a significant association of TV time and PC time with cardiometabolic function may be due to the relatively low TV time and PC time at this age; the mean TV time was 1.2 hour per day and mean PC time was 0.2 hours per day. This is confirmed by the significant association between excessive TV time (more than 2 hours per day) and waist circumference in our sample. Data were collected until December 2010. It is likely that PC time in 5–6 year olds has significantly increased since then due to the popularity of a tablet. To the best of our knowledge there are no temporal data on PC use in this age group but secular trends in older children suggest a marked increase in PC and Internet use in older children over time [22, 23]. As sedentary behaviours [24] as well as cardiometabolic biomarkers [15, 25] have been shown to track from childhood into adolescence, we recommend to examine a potential longitudinal association over longer time periods.

We believe it is important to distinguish between TV viewing and PC use for a number of reasons: i) PC use requires more muscle activity than TV viewing [26]; ii) TV and PC use might have different determinants and therefore likely require different intervention strategies to modify them; iii) TV and PC use differ in presence and nature of advertising and accompanying dietary behaviours; and iv) the social context in which TV and PC use occur mean that the mechanisms that underlie any associations with metabolic health might differ. This may explain our finding that PC time was significantly positively – i.e. beneficially - associated with HDLC. Similarly, Altenburg et al. found a significant inverse – i.e. beneficial - association of self-reported PC use with triglycerides and LDLC in obese adolescents [27]. Since the focus of the present study is on the independent effect of TV time and PC time we defined excessive TV time as > 2 hrs/day although this recommendation actually refers to ‘total entertainment screen-time’.

In contrast with our findings two previous studies found a significant association between TV time and blood pressure, one in children and one in adolescents. Martinez-Gomez et al. [12] found that TV viewing but not computer use was positively associated with blood pressure in US children aged 3 to 8 yrs old. However, in their study associations were not adjusted for birth weight, maternal education and PA levels. Gopinath et al. [28] found in 12–13 yr old Australian adolescents that TV time and playing video games, but not computer use was associated with diastolic blood pressure after adjusting for age, sex, ethnicity, parental education, height, BMI and PA. Ekelund et al. [13] found in older European children aged 9–10 and 15–16 yrs old a significant association between TV viewing and adiposity (sum of skinfolds). Similar to our findings, in their study, TV viewing was not associated with a similar obtained clustered cardiometabolic function score, after adjustment for PA, gender, age group, study location, sexual maturity, smoking status, birth weight, and parental socio-economic status. Saunders et al. [29] found that the relationship between screen time and health differed between 9 year old boys and girls. Leisure time computer/video game use was positively associated with continuous cardiometabolic risk and waist circumference, and negatively associated with HDL-cholesterol in boys only, while TV viewing was positively associated with continuous cardiometabolic risk, waist circumference and BMI Z-score in girls only. We found no effect modification by gender (data not shown), which may be explained by the younger age (5 versus 9 years) and lower TV (1.2 versus 2.0 hrs/day in boys and 1.1 versus 1.8 hrs/day in girls) and PC time (0.3 versus 1.1 hrs/day in boys and 0.2 versus 0.6 hrs/day in girls) in our sample.

A key strength of our study is the relatively large sample size and objective data on several continuous measures of cardiometabolic function. Another strength is the method of analysing several cardiometabolic risk factors together in a cardiometabolic function score, which is superior to analyses of individual risk factors in children. Furthermore, in contrast to previous studies that have typically focused on TV viewing only, we also included PC time. However, both measures were parent-reported and therefore subject to recall bias and social desirability bias. This may be an explanation for the relatively low reported TV and PC time. The question on PC time was on all PC use and may thus also have included active gaming. PA was also based on parent-report and the validity and reliability of the TV, PC and PA questions is unknown. Other limitations include the cross-sectional design, which precludes inferences about causality. Additionally, despite adjusting for several potential confounding factors, including PA, it is possible that other confounders may be relevant such as genotype and dietary habits. However, further adjustments for energy intake did not change the results (data not shown). Finally, the sample consisted of a majority of children of Dutch ethnicity compared to participating children without fasting blood sample (85% vs. 74%) limiting the generalizability of our findings.

Our study – as most previous studies on this topic – included fasting blood samples. Nowadays children are seldom in fasting state. Thus, whether sedentary time impairs the metabolic response to food in children is another important question. Therefore, we recommend that future studies examine the association between sedentary time and postprandial values of glucose, insulin and lipids.

## Conclusion

In conclusion, we found no convincing evidence for an association between TV or PC time, and cardiometabolic function in apparently healthy 5–6 yr olds. Since TV time and PC time increase with age, further study in prospective cohorts is recommended.

## Abbreviations

ABCD: Amsterdam Born Children and their Development
BMI: Body mass index
HDLC: High density lipoprotein cholesterol
LDLC: Low density lipoprotein cholesterol
PA: Physical activity
PC: Personal computer
TV: Television
WC: Waist circumference.
